# Blood volume-monitored regulation of ultrafiltration to decrease the dry weight in fluid-overloaded hemodialysis patients: a randomized controlled trial

**DOI:** 10.1186/s12882-017-0639-x

**Published:** 2017-07-17

**Authors:** Marlies Antlanger, Peter Josten, Michael Kammer, Isabella Exner, Katharina Lorenz-Turnheim, Manfred Eigner, Gernot Paul, Renate Klauser-Braun, Gere Sunder-Plassmann, Marcus D. Säemann, Manfred Hecking

**Affiliations:** 1 Department of Medicine III, Clinical Division of Nephrology & Dialysis, Medical University of Vienna, Vienna, Austria; 2 Nikkiso Europe GmbH, Hanover, Germany; 3 Section for Clinical Biometrics, Medical University of Vienna, Vienna, Austria; 4 1st Medical Department, Division of Dialysis, Kaiser-Franz-Josef Spital Vienna, Vienna, Austria; 5 3rd Medical Department, Division of Dialysis, SMZ-Ost Donauspital Vienna, Vienna, Austria

**Keywords:** Hemodialysis, Fluid overload, Blood volume monitoring

## Abstract

**Background:**

Because chronic fluid volume overload is associated with higher mortality, we tested whether blood-volume monitored regulation of ultrafiltration and dialysate conductivity (UCR) and/or regulation of ultrafiltration and temperature (UTR) would facilitate dry weight reduction, in comparison to conventional dialysis (CONV).

**Methods:**

We carried out a multicenter, 4-week, randomized controlled trial in hemodialysis patients ≥15% above normal extracellular fluid volume (ECV), per bioimpedance spectroscopy, who were randomized 1:1:1. Applying UCR (Nikkiso), UTR (Fresenius) and CONV, initial dry weight was reduced rapidly to target. Dry weight reduction was attenuated and eventually stopped at the occurrence of dialysis complications. The primary outcome was defined as intra- and postdialytic complications. Secondary outcomes were magnitudes of dry weight and blood pressure reduction.

**Results:**

Of 244 patients assessed, *N* = 95 had volume overload ≥15% above normal ECV. Fifty patients received the allocated interventions (*N* = 16 UCR, *N* = 18 UTR, *N* = 16 CONV) and completed the trial. The rate of complications was significantly lower in UTR compared to CONV (21 ± 21% vs 34 ± 20%, *p* = 0.022), and also compared to UCR (vs 39 ± 27%, *p* = 0.028), but not statistically different between UCR and CONV (*p* = 0.93). Dry weight reduction was significantly higher in UTR compared to UCR (5.0 ± 3.4% vs 2.0 ± 2.7% body weight, *p* = 0.013), but not compared to CONV (vs 3.9 ± 2.1% body weight, *p* = 0.31). Systolic blood pressure reduction throughout the intervention phase was 17 ± 22 mmHg overall, but not significantly different between the three groups. Average maximum ultrafiltration rates were significantly higher in UTR than in UCR and CONV, at statistically similar dialysis times. Retrospective examination of randomly selected hemodialysis sessions in the UCR group identified technical mistakes in 36% of the dialysis sessions, despite considerable training efforts.

**Conclusions:**

Even in patients with volume overload, fluid removal was challenging. Despite the relative advantage of UTR, which must be interpreted with caution in view of the poor technical execution of UCR, this study renders clear that fluid removal must not be reinforced rapidly. Apprehension of this obstacle is imperative for future clinical and academic endeavors aimed at improving dialysis outcomes by correcting volume status.

**Trial registration:**

ClinicalTrials.gov (NCT01416753), trial registration date: August 12, 2011.

## Background

In patients with end-stage renal disease undergoing maintenance hemodialysis treatment, excessive fluid volume is a major risk factor for death [1, 2]. Fluid overload, however, is potentially modifiable, and dialysis centers with a strong emphasis on strict volume control consistently report excellent survival results [3, 4]. In a observational study, the mortality benefit in hemodialysis patients without fluid overload was independent of blood pressure and dietary salt restriction [5], indicating that the dialysis prescription should primarily be focussed on the ideal target ‘dry’ weight for every patient.

The dry weight concept has evolved over time [6], and nowadays incorporates objective measures of volume status [7]. Combining whole-body bioimpedance spectroscopy with a body composition model enables the quantification of fluid overload (in liters and in %) above the extracellular fluid volume (ECV) presumed ‘normal’ in a healthy individual [8]. Despite their impaired renal function, hemodialysis patients can thus be classified as fluid overloaded, and their optimal dry weight can be prescribed precisely, based on the bioimpedance spectroscopy measurement. This approach has been validated against gold standard methods of fluid assessment [9] including high-quality clinical assessment, demonstrating that it is an important addition to clinical judgement.

Strategies to facilitate fluid removal include regulation of ultrafiltration based on blood volume monitoring (BVM) and concurrent modification of dialysate conductivity [10–12] as well as cooling [13, 14]. These techniques have shown to improve hemodynamic stability during dialysis [15, 16]. In a large prospective trial (CLIMB), however, the availability of a BVM device increased mortality and failed to improve intradialytic complications, such as hypotension and muscle cramps [17]. Importantly, CLIMB and other studies on dialysis techniques did not exclusively include fluid overloaded patients, who might benefit most from BVM-based hemodialysis regulation. In the present randomized multicenter trial, we therefore investigated in volume-overloaded hemodialysis patients only, whether regulation of ultrafiltration and dialysate conductivity (UCR) and/or regulation of ultrafiltration and temperature (UTR) decreases dialysis complications upon systematic dry weight reduction [18]. An important secondary objective was to determine if UCR and/or UTR would allow greater dry weight reduction than CONV. We specifically aimed at putting UCR and/or UTR to the test, by lowering the dry weight according to the fastest published algorithm [19], which was dynamically slowed down upon occurrence of dialysis-related complications.

## Methods

### Study algorithm

The study protocol was peer reviewed and published in detail before study initiation [18]. It was approved by two independent local ethics committees (Medical University of Vienna, EK#365/2011; City of Vienna, EK-11-222-1211) and registered at ClinicalTrials.gov (NCT01416753). Prior to study start, three maintenance hemodialysis facilities evaluated the volume status in all patients, using the Body Composition Monitor (BCM, Fresenius Medical Care, Germany; details in [20]). All measurements were carried out after a short interdialytic interval. Patients ≥18 years who presented with predialysis fluid overload ≥15% above normal ECV (corresponding roughly to 2.5 L of excess fluid in a typical hemodialysis patient [1]) were eligible for study participation, unless they had been dialyzing <3 months. Charlson Comorbidity Index was calculated as previously described [20].

The study participants’ ideal dry weight was calculated as follows: Predialysis body weight [kg] – (fluid overload +7% ECV [in L, set equal to kg]). Dry weight reduction from initial to ideal dry weight followed the algorithm of the ‘Dry-Weight Reduction in Hypertensive Hemodialysis Patients’ (DRIP)-trial [19]. In summary, initial dry weight was lowered in steps of 0.1 kg/10 kg body weight at every hemodialysis session, unless the patient had intra- or postdialytic complications (described below). At the occurrence of complications, dry weight reduction was stopped and the additional weight loss was reduced by 50% at the next session. This process was repeated until even a dry weight reduction of 0.2 kg was no longer tolerated.

Prior to study start, complications were subclassified as follows: [1] intradialytic cramping; [2] intradialytic hypotension, defined as >40 mmHg drop in systolic blood pressure [SBP] within 30 min (clinically asymptomatic); [3] intradialytic hypotension, defined as >40 mmHg SBP within 30 min (clinically symptomatic by patient-reported lightheadedness, dizziness and/or fainting); [4] intradialytic hypotension, defined as an unspecified drop in SBP (clinically symptomatic); [5] all forms of intradialytic hypotension ([2], [3] or [4]), together; [6] unspecified intradialytic complication, most likely related to fluid withdrawal; and [7] patient-reported postdialysis complication, most likely related to fluid withdrawal (=unspecified postdialytic complication). The rate of complications was determined for the above categories [1–7], as well as for all complications together by dividing “per number of hemodialysis sessions at risk”, i.e. per all hemodialysis sessions of this study (intention-to-treat [ITT] analysis), as well as all per hemodialysis sessions with dry weight reduction (per protocol [PP] analysis).

### BVM regulation technique

Patients on hemodiafiltration protocols were switched to hemodialysis, but treatment time, prescribed dialysate sodium concentrations and blood flow rates remained unchanged after randomization. The principles of the dialysis techniques under investigation (UCR and UTR), compared to the standard (CONV), have been thoroughly discussed [18]. Briefly, with blood volume monitoring, ultrafiltration rates are variable and constantly adapted during the hemodialysis session.

UTR was executed using Fresenius dialysis machines, model 5008, with the “BVM-regulation mode” switched on as well as the “BTM body temperature regulation” activated. UCR was executed using Nikkiso dialysis machines, model DBB 05, with the “blood volume UF control” activated and the “blood volume conductivity control (BV-COC)” activated. Both systems require a monitoring period, wherein the Fresenius 5008 uses ultrasound to measure “relative” blood volume (“RBV”) changes and wherein the Nikkiso DBB 05 uses optical conductivity to monitor “differences” in blood volume (“dBV”). Blood volume data were therefore generated during several hemodialysis sessions prior to study start (run-in period), in order to set the “critical RBV” for every patient using the Fresenius 5008 as well as the “dBV reference curve” for every patient using the Nikkiso DBB 05, respectively. While the critical RBV has to be set manually (i.e. the critical RBV is at least that RBV during which the patient has not had dialysis complications throughout previous treatments), the dBV reference curve is determined by the DBB 05 itself, through the steepness and the calculated dBV final value using the data generated at the previous sessions.

Briefly on temperature regulation using the BTM in the Fresenius 5008, the body temperature is kept constant when “BTM body temperature regulation” is activated. Briefly on conductivity regulation using the Nikkiso DBB 05, the total sodium that can be transferred during the hemodialysis session is defined by the patient’s prescribed dialysate sodium, which transfers into the selection of a specific conductivity (for example 14 mS for DNa 138 mEq/L). When “blood volume conductivity control (BV-COC)” is activated in the DBB 05, the machine responds to the change in the actual dBV curve during the dialysis session, by a pre-defined algorithm. Importantly, the total dialysate sodium available during the hemodialysis session is always constant (i.e. conductivity multiplied by dialysis time in hours).

With “CONV”, ultrafiltration rates were constant during the hemodialysis session, and determinable by dividing the total amount of ultrafiltration by the treatment time. The duration of the intervention phase was 12 hemodialysis sessions, starting immediately after the run-in period for the UTR and UCR groups, and immediately after randomization for the CONV group. Besides the UTR group, dialysate temperature remained constant at 36.5 °C throughout treatments, similarly to dialysate sodium in groups other than the UCR group, where the standard setting was 138 mmol/L.

### Withdrawal of antihypertensives

As pre-specified before study start [18], withdrawal of antihypertensives was started 1 week prior to the first hemodialysis session with reduced dry weight. Alpha and beta blockers were withdrawn first, followed by calcium channel blockers and blockers of the renin-angiotensin aldosterone system last. Withdrawal of antihypertensives was aimed at being identical throughout the study groups.

### Sample size calculation, randomization and statistical analyses

In the conventional arm of the previous CLIMB-study [17], dialysis-related complications occurred 14 times per 100 patient days at risk. Based on this rate and an expected standard deviation of 10% between groups, alpha =0.05 and beta =0.2, a sample size of 17 patients per group was determined to detect a minimum difference of 10% between groups [18]. Prior to study start, we planned to enroll 20 patients in each arm, because we expected a drop-out rate of 10%, and because patients could not be replaced.

The code for center-stratified block randomization into three study groups at 1:1:1 ratio was developed using an internet based randomization tool [21], and not revealed to the investigators prior to having obtained written informed consent of all participating patients. Patients were then randomized all at once at every center, in the order of the investigator’s usual rounding at the dialysis facility. Patients.

Data were displayed descriptively as means ± standard deviation (SD), medians (and interquartile range [IQR]), absolute and relative frequencies. Intention-to-treat and per-protocol (only dialysis session with successful dry weight reduction) analyses were performed. Models for count data were used to evaluate the overall effect of the dialysis method on the number of complications during dialysis sessions: Poisson regression was used if the outcome followed a Poisson distribution, otherwise the more general negative binominal regression was applied. The decision between the two models was based on Akaike’s information criterion. To account for different numbers of dialysis sessions among the patients, its logarithm entered the models as offset variable. First, a global *p*-value for the comparison of the dialysis methods with regard to their effect on the outcome was derived from the model. If this global test was significant then further pairwise contrasts between the three study groups were estimated. To account for multiple comparisons, the overall effect tests derived from the models of complication categories [1–6] were corrected using the Bonferroni-Holm method [22]. In the case of a small number of complications in a study group it was necessary to employ a Firth-corrected Poisson regression model [23] to prevent the occurrence of infinite parameter estimates. For outcome measures which involve comparisons of values from the start and end of the study an analysis of covariance (ANCOVA) was performed, with the dialysis method entering as independent variable and the baseline value as covariate. If necessary, the influence of a non-normal distribution of residuals was assessed by conducting an ANCOVA on ranks. The effect of outliers was reduced by winsorising the data. For all other outcome measures, overall differences of the three methods were determined by analysis of variance (ANOVA). If an overall *p*-value from an ANCOVA or ANOVA was significant, further comparisons were made between the three study groups by testing UCR and UTR against CONV and one against the other. The unpaired, two-tailed t-test was used for comparison of all normally distributed, continuous variables (also baseline characteristics). Non-normally distributed variables were analyzed by Mann-Whitney U test. Categorical variables were analyzed using the unadjusted chi-square test or Fisher exact test, when appropriate. Calculations were performed using MS Excel 2010, SAS 9.3 for Windows, as well as R 3.0.2 for the Firth-corrected Poisson regression models.

### Study duration

This study originally had a crossover design with 3 intervention phases (12 hemodialysis sessions each), enabling all participants to use the 3 dialysis techniques [18]. Because patients were not required to revert to their fluid overloaded state after crossing over to another treatment modality, the crossover design solely served the purpose of exploring additional endpoints, for example which method would patients choose as their preferred hemodialysis treatment. The primary outcome, and most secondary outcome measures as well as the sample size calculation were independent of the other intervention phases, however. Because of the high rate of dialysis-related complications (described in the Results section), the study was terminated after the first intervention phase.

## Results

### Volume assessment and study participants

**Fig. 1:**
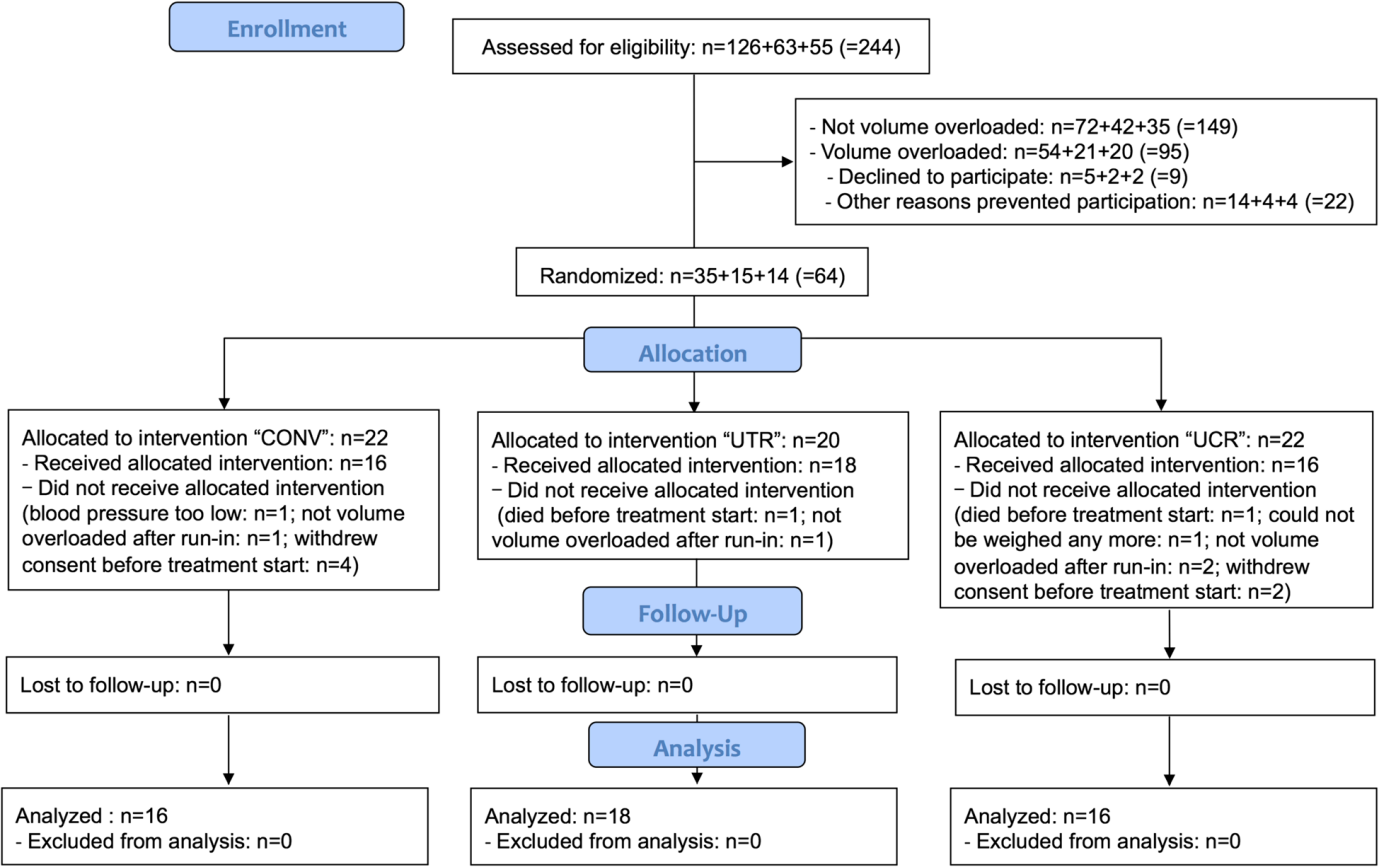
Enrollment, randomization, and follow-up of study participants. CONSORT (Consolidated Standards of Reporting Trials) flow chart of the study

We assessed the predialysis volume status in 244 hemodialysis patients. 95 patients (39%) had predialysis fluid overload ≥15% above normal ECV [20]. The fluid overload reading was in accordance with clinical judgement in all patients. Sixty-four of all fluid overloaded patients agreed to participate and were randomized. The most common reason that precluded study participation, other than declining (*N* = 9 patients, 9%) was a patient’s intellectual inability to understand the study design (*N* = 13 patients, 14%), which was worsened by a language barrier despite attempts to provide translation in 6 patients and prevented the investigators from obtaining written informed consent. Fourteen patients left the study during the run-in period before receiving any of the allocated interventions, 6 of them at their own discretion, 4 of them because they were not fluid overloaded anymore after the run-in period, and the remaining 4 because they either died, because blood pressure was too low or because they could not be weighed any more (Fig. 1). All 50 participants who received the allocated interventions (CONV, UTR, UCR) finished the study. Baseline demographics and patient characteristics were well balanced between the three study groups, with the exception of dialysis vintage, which was lower in the UTR group (Table 1). Charlson Comorbidity Index was comparable between the three analyzed groups: 4.6 ± 1.9 (CONV) vs. 5.8 ± 3.3 (UTR) vs. 4.2 ± 1.8 (UCR, *p* = 0.306).

**Table 1:** Baseline demographics and patient characteristics

	CONV	UTR	UCR	p^+^
N participants	16	18	16	
Male [%]	63	61	56	0.93
Center 1 = MUV [%]	69	61	63	0.89
Center 2 = KFJ [%]	6	22	19	0.44
Center 3 = SMZ [%]	25	17	19	0.83
Age [years]	63 ± 13	62 ± 16	61 ± 17	0.94
Time on Dialysis [years]	3.1 ± 2.5	1.9 ± 1.7	3.2 ± 1.8	0.10
Height [cm]	174 ± 11	169 ± 7	170 ± 12	0.38
Body Mass Index [kg/m^2^]	24.7 ± 6.1	24.3 ± 4.6	22.6 ± 4.4	0.48
No Antihypertensives [%]	0	17	0	0.06
-Alpha Blockers [%]	50	33	31	0.61
-Beta Blockers [%]	81	56	69	0.39
-ACEI/ARB [%]	56	56	31	0.58
-Calcium Channel Blockers [%]	56	50	63	0.29
-Vasodilators [%]	25	17	44	0.66
-‘Central’ Antihypertensives [%]	25	28	31	0.42
-Diuretics [%]	25	28	25	0.71
Central Venous Catheters [%]	50	56	25	0.17
Hemodiafiltration [%]^++^	31	28	31	0.97
Ethnicity				
Caucasian [%]	100	89	94	0.41
African/Black [%]	0	0	6	0.35
Asian [%]	0	11	0	0.16
Type 1 DM [%]	0	6	0	0.46
Type 2 DM [%]	31	28	25	0.93
Kidney Disease				
Vascular [%]	19	0	19	0.15
Glomerular [%]	13	22	19	0.77
Polycystic [%]	6	0	13	0.32
Tubulointerstitial [%]	13	22	13	0.68
Diabetic Glomerulosclerosis [%]	25	17	19	0.83
Unknown [%]	25	39	19	0.42

*CONV* conventional hemodialysis, *UTR* ultrafiltration and temperature regulation, *UCR* ultrafiltration and dialysate conductivity regulation, *N* number, *DM* diabetes mellitus

ITT analysis. ^+^Overall *p*-values determined by analysis of variance. ^++^All other patients on hemodialysis

### Dry weight and blood pressure reduction

All information on volume Status at baseline, dry weight reduction and blood pressure can be found in (Tables 2, 3, 4, 5 and 6). The dry weight reduction process was unsuccessful in 7 of the 50 study participants, because several patients did not allow the nursing personnel to lower their dry weight due to subjective body image issues (*N* = 3), or because their nurses and doctors consistently felt that it was not in the patients’ best interest to do so (*N* = 4), although the results of the volume assessment were not considered false. Importantly, such a judgment occurred at all 3 centers independently, with similar rationale provided. Predialysis fluid overload was similar between the three study groups and averaged to 4.8 ± 1.6 L (23.9 ± 5.1% ECV), over all 3 groups. In the UTR group, the dry weight reached by the end of the intervention phase was the lowest (−5.0 ± 3.4% body weight), and significantly lower than in UCR (−2.0 ± 2.7% body weight, *p* = 0.013, Tables 2, 3, 4, 5 and 6), but not significantly lower than in CONV (−3.9 ± 2.1% body weight, *p* = 0.31, Tables 2, 3, 4, 5 and 6). From the beginning to the end of the dry weight reduction process, SBP decreased markedly, by 17 ± 22 mmHg overall, with no significant differences observed between the three study groups. DBP also decreased, by 7 ± 20 mmHg over all 3 study groups from the beginning to the end of the dry weight reduction process, but remained slightly higher in the UCR group, compared to UTR and CONV. The number of antihypertensive agents withdrawn per patient was 0.8 ± 1.0, over all 3 study groups, and was higher in CONV than in UTR and UCR.

**Table 2:** Weight, fluid overload and blood pressure at baseline

				*p*-value^+^
	CONV	UTR	UCR	Overall
N participants	16	18	16	
Body Weight pre hemodialysis [kg]	75.7 ± 23.5	69.2 ± 11.3	66.4 ± 17.0	0.32
Initial Dry Weight [kg]	74.7 ± 23.2	67.4 ± 11.6	65.2 ± 16.8	0.30
Ultrafiltration (L)	1.1 ± 0.9	1.8 ± 1.0	1.5 ± 1.0	0.10
Fluid Overload pre hemodialysis [L]	4.6 ± 1.4	5.3 ± 1.7	4.3 ± 1.7	0.18
ECV [L]	20.4 ± 4.4	19.2 ± 2.9	18.5 ± 4.4	0.42
Fluid Overload pre hemodialysis [% ECV]	22.9 ± 5.2	25.4 ± 4.6	23.2 ± 5.4	0.30
Fluid Overload post hemodialysis [% ECV]	17.2 ± 7.8	15.2 ± 8.1	14.5 ± 7.8	0.60
SBP-pre [mmHg]	146 ± 30	136 ± 21	148 ± 21	0.34
DBP-pre [mmHg]	76 ± 16	68 ± 13	76 ± 20	0.26

*CONV* conventional hemodialysis, *UTR* ultrafiltration and temperature regulation, *UCR* ultrafiltration and dialysate conductivity regulation, *N* number, *ECV* extracellular volume, *SBP* systolic blood pressure, *DBP* diastolic blood pressure, *BCM* body composition monitor

ITT analysis ^+^ *P*-values between groups determined by two-tailed t-test, and Mann-Whitney U test. Overall *p*-values determined by analysis of variance and analysis of covariance (for comparison of repeated measures). In one patient from the UTR group only absolute fluid overload values could be measured; relative fluid overload values could not be calculated by the BCM device due to technical issues, resulting in deviation of the FO value from ECV

**Table 3:** Group-wise dry weight reduction

				*p*-value^+^			
	CONV	UTR	UCR	CONV vs UTR	CONV vs UCR	UTR vs UCR	Overall
N participants with dry weight reduction	12	17	14				
Initial Dry Weight [kg]	75.6 ± 26.2	67.6 ± 11.9	66.9 ± 16.6				0.44
Ideal Dry Weight [kg] (=Normohydration Weight - 7% ECV)	70.3 ± 25.6	63.2 ± 11.2	62.9 ± 16.0				0.49
Diff. from Initial Dry Weight [kg]	−5.3 ± 1.8	−4.3 ± 1.8	−4.0 ± 2.0				0.27
[% Body Weight]	−7.4 ± 2.2	−6.4 ± 2.3	−5.7 ± 2.7				0.22
Dry Weight Reached [kg]	72.6 ± 25.3	64.1 ± 10.7	65.4 ± 16.6				0.42
Diff. from Initial Dry Weight [kg]	−3.0 ± 1.9	−3.5 ± 2.8	−1.5 ± 1.9	0.30	0.15	0.010	0.036
[% Body Weight]	−3.9 ± 2.1	−5.0 ± 3.4	−2.0 ± 2.7	0.31	0.06	0.013	0.022
Ideal Dry Weight Missed by … [kg]	2.3 ± 1.8	0.8 ± 2.1	2.5 ± 1.8	0.06	0.87	0.031	0.044
[% Body Weight]	3.5 ± 2.7	1.4 ± 3.2	3.7 ± 2.4				0.06

*CONV* conventional hemodialysis, *UTR* ultrafiltration and temperature regulation, *UCR* ultrafiltration and dialysate conductivity regulation, *N* number, *ECV* extracellular volume, *SBP* systolic blood pressure, *DBP* diastolic blood pressure, *BCM* body composition monitor

PP analysis. ^+^ *P*-values between groups determined by two-tailed t-test, and Mann-Whitney U test. Overall *p*-values determined by analysis of variance and analysis of covariance (for comparison of repeated measures). In one patient from the UTR group only absolute fluid overload values could be measured; relative fluid overload values could not be calculated by the BCM device due to technical issues, resulting in deviation of the FO value from ECV

**Table 4:** Group-wise course of systolic blood pressure

				*p*-value^+^
	CONV	UTR	UCR	Overall
N participants with dry weight reduction	12	17	14	
SBP-pre: BCM Measurement [mmHg]	142 ± 27	136 ± 21	148 ± 21	0.28
SBP-pre: Start Intervention Phase [mmHg]	146 ± 19	143 ± 26	154 ± 27	0.45
SBP-post: Start Intervention Phase [mmHg]	133 ± 22	132 ± 17	144 ± 31	0.34
SBP-pre: End Intervention Phase [mmHg]	127 ± 20	127 ± 15	136 ± 13	0.26
SBP-post: End Intervention Phase [mmHg]	112 ± 16	117 ± 15	129 ± 22	0.06
Delta SBP-pre: Start-to-End In Phase [mmHg]	−16 ± 23	−16 ± 17	−19 ± 27	0.43
Delta SBP-post: Start-to-End In Ph. [mmHg]	−19 ± 19	−16 ± 11	−15 ± 31	0.27

*CONV* conventional hemodialysis, *UTR* ultrafiltration and temperature regulation, *UCR* ultrafiltration and dialysate conductivity regulation, *N* number, *ECV* extracellular volume, *SBP* systolic blood pressure, *DBP* diastolic blood pressure, *BCM* body composition monitor

PP analysis. ^+^ *P*-values between groups determined by two-tailed t-test, and Mann-Whitney U test. Overall *p*-values determined by analysis of variance and analysis of covariance (for comparison of repeated measures). In one patient from the UTR group only absolute fluid overload values could be measured; relative fluid overload values could not be calculated by the BCM device due to technical issues, resulting in deviation of the FO value from ECV

**Table 5:** Group-wise course of diastolic blood pressure

				*p*-value^+^
	CONV	UTR	UCR	Overall
N participants with dry weight reduction	12	17	14	
DBP-pre: BCM Measurement [mmHg]	74 ± 15	67 ± 13	76 ± 20	0.23
DBP-pre: Start Intervention Phase [mmHg]	73 ± 14	70 ± 15	85 ± 26	0.09
DBP-post: Start Intervention Phase [mmHg]	70 ± 13	66 ± 13	75 ± 14	0.17
DBP-pre: End Intervention Phase [mmHg]	66 ± 11	64 ± 11	76 ± 13	0.02
DBP-post: End Intervention Phase [mmHg]	57 ± 15	63 ± 12	69 ± 14	0.10
Delta DBP-pre: Start-to-End In Ph. [mmHg]	−7 ± 11	−5 ± 12	−8 ± 31	0.10
Delta DBP-post: Start-to-End In Ph. [mmHg]	−12 ± 14	−3 ± 9	−6 ± 14	0.14

*CONV* conventional hemodialysis, *UTR* ultrafiltration and temperature regulation, *UCR* ultrafiltration and dialysate conductivity regulation, *N* number, *ECV* extracellular volume, *SBP* systolic blood pressure, *DBP* diastolic blood pressure, *BCM* body composition monitor

PP analysis. ^+^ *P*-values between groups determined by two-tailed t-test, and Mann-Whitney U test. Overall *p*-values determined by analysis of variance and analysis of covariance (for comparison of repeated measures). In one patient from the UTR group only absolute fluid overload values could be measured; relative fluid overload values could not be calculated by the BCM device due to technical issues, resulting in deviation of the FO value from ECV

**Table 6:** Course of amount of antihypertensive medication

				*p*-value^+^
	CONV	UTR	UCR	Overall
Among N particips. w. dry weight reduction	12	17	14	
Antihypertensives per Pat: BCM Meas’ment	3.3 ± 1.4	2.9 ± 2.1	3.5 ± 1.9	0.65
Antihypertensives per Pat: DW reduct. End	2.2 ± 0.8	2.4 ± 2.0	2.9 ± 1.6	0.73
Antihypertensives withdrawn per Pat	1.6 ± 1.1	0.5 ± 1.0	0.4 ± 0.7	0.08

*CONV* conventional hemodialysis, *UTR* ultrafiltration and temperature regulation, *UCR* ultrafiltration and dialysate conductivity regulation, *N* number, *ECV* extracellular volume, *SBP* systolic blood pressure, *DBP* diastolic blood pressure, *BCM* body composition monitor

PP analysis. ^+^ *P*-values between groups determined by two-tailed t-test, and Mann-Whitney U test. Overall *p*-values determined by analysis of variance and analysis of covariance (for comparison of repeated measures). In one patient from the UTR group only absolute fluid overload values could be measured; relative fluid overload values could not be calculated by the BCM device due to technical issues, resulting in deviation of the FO value from ECV

### Dialysis-related complications (primary endpoint)

**Fig. 2:**
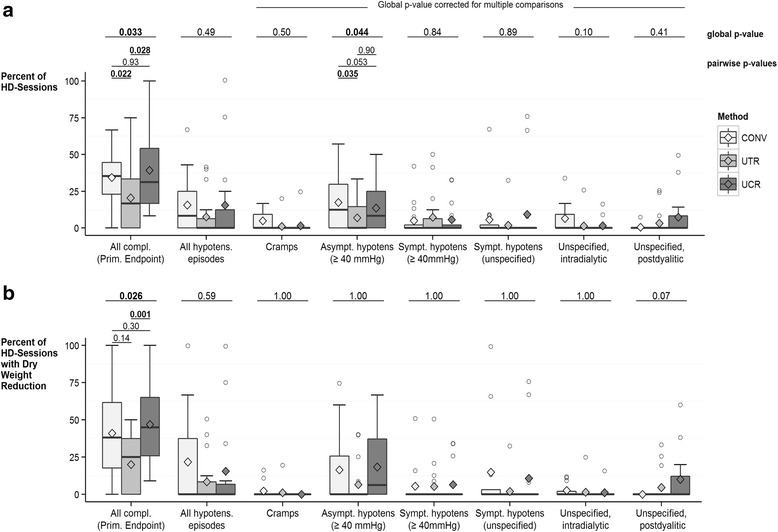
Dialysis-Related Complications. The number of complications that a particular patient had during the intervention phase were divided by the number of his/her hemodialysis sessions at risk, i.e. all sessions during the intervention phase (panel **a**, *N* = 50 patients: *N* = 16 in CONV, *N* = 18 in UTR, *N* = 16 in UCR), or only the hemodialysis sessions with dry weight reduction during the intervention phase (panel **b**, *N* = 43 patients: *N* = 12 in CONV, *N* = 17 in UTR, *N* = 14 in UCR). The resulting percentages are depicted as boxplots including the means (shown as diamond). *P*-values shown above the boxplots for each complication outcome were derived from models for count data. Pairwise *p*-values are only shown if the global *p*-value was significant

The average rate of complications over all 3 study groups was 31 ± 24% during the entire invention phase (Fig. 2a), and 35 ± 27% when considering only those hemodialysis session where the dry weight was reduced (Fig. 2b). Both rates were lower in the UTR group, compared to CONV and UCR (CONV 34 ± 20% and 41 ± 30%; UTR: 21 ± 21% and 20 ± 19%; UCR: 39 ± 27% and 47 ± 27%). Statistical significance was reached in the group comparison UTR versus UCR when considering all hemodialysis sessions as well as hemodialysis sessions with dry weight reduction (Fig. 2a/b). UTR versus CONV was statistically significant when considering all hemodialysis sessions (Fig. 2a).

### Ultrafiltration rates and quality control

During the intervention phase, the average ultrafiltration rate over all groups was 609 ± 255 mL/h (or 5.6 ± 4.0 mL/h/kg body weight), with higher average ultrafiltration rates observed in UCR, compared to either CONV or UTR (Table 7). During the intervention phase, we documented the ultrafiltration rates every 30 min during all hemodialysis sessions and recorded the lowest as well as highest value. In the UTR group, the average of all highest ultrafiltration rates was significantly higher than in UCR, while the ultrafiltration rates in CONV had been kept constant per the study’s protocol, such that there was no difference between highest and lowest rates (Table 7).

**Table 7:** Ultrafiltration rates, blood flow rates and dialysis time

				*p*-value^+^			
	CONV	UTR	UCR	CONV vs UTR	CONV vs UCR	UTR vs UCR	Overall
N participants with dry weight reduction	12	17	14				
Average UF rate during study [mL/h]	429 ± 184	583 ± 218	745 ± 200	0.06	0.001	0.042	0.002
Average UF rate during study (ml/h/kg body weight)	6.5 ± 3.7	9.0 ± 3.9	11.5 ± 3.7	0.104	0.003	0.082	0.009
Average UF rate low during Intervention Phase [mL/h]	622 ± 149	479 ± 132	476 ± 124	0.018	0.018	0.955	0.020
Average UF rate low during Intervention Phase (ml/h/kg body weight)	8.9 ± 3.5	7.2 ± 2.2	7.6 ± 2.3	0.149	0.308	0.645	0.286
Average UF rate high during Intervention Phase [mL/h]	657 ± 186	1209 ± 292	873 ± 244	<0.01	0.008	0.004	<0.01
Average UF rate high during Intervention Phase (ml/h/kg body weight)	8.9 ± 3.5	18.2 ± 5.4	13.9 ± 5.4	<0.01	0.016	0.054	<0.01
BF rate throughout [mL/min]	290 ± 29	281 ± 37	278 ± 27				0.61
Dialysis time [h/session]	4.0 ± 0.4	3.9 ± 0.3	4.0 ± 0.6				0.90

*CONV* conventional hemodialysis, *UTR* ultrafiltration and temperature regulation, *UCR* ultrafiltration and dialysate conductivity regulation, *N* number, *UF* ultrafiltration, *BF* blood flow

Per protocol analysis. ^+^ *P*-values between groups determined by two-tailed t-test, and Mann-Whitney U test. Overall *p*-values determined by ANOVA

One hundred and twelve randomly selected hemodialysis sessions in the UCR group were retrospectively examined, in order to judge the execution of the dialysis technique. Mistakes in the execution of the dialysis technique occurred in 40 hemodialysis sessions (36%). The principal mistake was having either the conductivity regulation and/or the regulation of ultrafiltration turned off at treatment start, which occurred in 20 hemodialysis sessions. In 8 hemodialysis sessions, the reference line was unavailable, and in 6 hemodialysis sessions, the reference line was not adapted in order for the patient to reach his/her prescribed dry weight, although the patient’s blood pressure would have allowed this procedure.

## Discussion

In the present randomized controlled trial, we investigated whether rapid dry weight reduction can be achieved with fewer dialysis-related complications when using the techniques provided by modern hemodialysis machines. The rate of complications was significantly lower in UTR compared to CONV. However, the high rate of dialysis-related complications, in spite of the fact that fluid overload was bioimpedance spectroscopy-proven, is concerning, and several aspects of this ‘real-world study’ should be critically discussed.

First, it was an unfortunate but not unexpected study limitation that 4 study patients of all 64 who were initially randomized (6%) were not fluid overloaded after the run-in period, indicating that they might have understood to lower their dry weight independently of the study protocol. Among those 50 who did start with the allocated interventions (see Fig. 1), 7 participants (14%) did not undergo dry weight reduction. Because none of them refused the allocated interventions (CONV, UTR, or UCR), they were not considered study drop-outs, which allowed assessing dialysis-related complications, but separately analyzing all hemodialysis sessions with dry weight reduction versus without.

We found that 1 out of 3 hemodialysis sessions was accompanied by a complication, predominantly hypotensive episodes and cramps, indicating that this concern was not unjustified. This rate was higher than in the CLIMB-study (14%) [17], but comparable to the DRIP-trial, where the proportion of treatments complicated by cramps rose up to 22.0% in the ultrafiltration group, and the proportion of treatments complicated by hypotension rose up to 24.8% [19]. Thus, our study prerequisite of including fluid overloaded patients only did not prevent a relatively high complication rate, and provides evidence for the importance of reducing the dry weight more slowly [24].

The high rate of complications also provides the reason why our study was terminated after the first intervention phase, rather than being continued according to the original cross-over study design. While the failure to carry out the initial crossover design is a study limitation, we previously acknowledged that this design served the purpose of secondary endpoint evaluation only. The reason is that patients could not reasonably have been requested to revert to their previous degree of fluid overload after each study phase [18]. A second BCM measurement at the end of study phase 1 is lacking, which represents a study limitation.

Fewer complications were observed in the UTR group, indicating that UTR was the superior technique in the present setting. One might speculate that UTR might have exerted a more rapid effect than UCR because of the use of the body temperature monitor [13, 14, 25], which rendered the investigated dialysis techniques principally different, and exerts a mechanism that is well known to prevent intradialytic hypotension [26]. However, caution must be applied, as some baseline differences between UTR and the other groups remained despite randomization and actual body and dialysis temperature were not recorded throughout the study. For example, ultrafiltration rates and dialysis vintage were higher in UCR, despite similar Charlson Comorbidity Index and similar dialysis catheter rates. The limited sample size of the present study hampers precluding a type 1 error, which is, however, rendered unlikely by the substantial magnitude of the difference in complications between UCR and UTR.

Yet, importantly, UTR might have been easier to use than UCR, as indicated by the high rate of mistakes observed in the UCR group, and corroborated by the verbal feed-back of the nursing personnel. Although personal training sessions had taken place repeatedly, they might not have been sufficient to ensure proper execution of the technique itself. If the training standards were insufficient at all three of our study centers though, it seems rational to assume that BVM might likewise be a poorly understood technique at other facilities as well, and especially those facilities that are not conducting an academic study. Clearly, the operator convenience of any BVM-based technique will potentially limit its own use. Due to these operator-specific pitfalls as well as other limitations, these techniques should be used with caution [27]. Our study, however, does not allow conclusions on other UCR-like systems, such as Hemocontrol by Gambro, or others [28, 29]. Furthermore, the execution difficulties of the UCR sessions that our personnel encountered make a conclusion on the efficacy of this system difficult.

The “ideal” dry weight targets based on the BCM measurement have, to the best of our knowledge, not yet been determined, thereby remaining an important field for further research. In the process of developing this study’s protocol, the study team decided to set the ideal dry weight even below the target that results from the degree of fluid overload subtracted from the predialysis weight (by 7% ECV) [18]. This procedure relies on the clinical logic that patients who leave the hemodialysis session in slight hypovolemia will reach normovolemia after subsequent fluid intake, and a state of fluid overload only thereafter, indicating that their time-averaged fluid overload will be closer to healthy people. Our strategy allowed us not only to be in accordance with clinical reasoning, but also to lower the dry weight so considerably that we could expect clearer study results, i.e. greater differences in complications from the dry fluid removal process.

This study is limited by lacking data on orthostatic blood pressure postdialysis. Concomitant with dry weight reduction, significantly lower predialysis blood pressure values were attained in all 3 patients groups, even though a reduction of the antihypertensive therapy had occurred per the study protocol, with approximately one antihypertensive agent eliminated per patient. Although observational studies have shown that the mortality risk associated with blood pressure begins to increase at levels that are higher than in the general population, while lower blood pressure confers a greater mortality risk [30–32], dialysis patients with close to normal fluid status may have low blood pressure levels - but still better survival rates - than those with fluid overload [5]. Being able to reduce the number of antihypertensive agents through greater fluid removal therefore seems desirable, for patient convenience but presumably also for patient outcomes. Further, an explorative assessment of pre- and post-dialysis serum sodium in UCR-treated patients would enable future insights into blood pressure regulation, responsiveness to dialysate sodium adaptation and potential benefits of this treatment strategy.

## Conclusions

In conclusion, the results of the present randomized, controlled trial show that even in patients with significant fluid overload, fluid removal is challenging. Despite the apparent technical advantage of UTR, dry weight reduction cannot be recommended to be reinforced rapidly with either supporting technique, due to the high rate of intra- and postdialytic complications. Furthermore, although many observational analyses have shown that patients without chronic fluid overload have better survival [1–5], future studies have yet to confirm that the correction of volume status prospectively improves outcomes [33]. To facilitate such endeavors, the present data may become valuable tools to design the most appropriate strategies for fluid removal.

## Abbreviations

ANCOVA: Analysis of covariance
ANOVA: Analysis of variance
BCM: Body composition monitor
BTM: Blood temperature monitor
BVM: Blood volume monitoring
COC: Conductivity control
CONV: Conventional hemodialysis
DBP: Diastolic blood pressure
dBV: Differences in blood volume
DNa: Dialysate sodium
ECV: Extracellular fluid volume
IQR: Interquartile range
ITT: Intention to treat
PP: Per protocol
SBP: Systolic blood pressure
UCR: Ultrafiltration and dialysate conductivity regulation
UTR: Ultrafiltration and dialysate temperature regulation
